# Volatile oil of *Acori tatarinowii rhizoma*: potential candidate drugs for mitigating dementia

**DOI:** 10.3389/fphar.2025.1552801

**Published:** 2025-04-23

**Authors:** Yifan Bu, Songzhe Li, Ting Ye, Yuqing Wang, Mingrong Song, Jing Chen

**Affiliations:** ^1^ College of Basic Medical and Sciences, Heilongjiang University of Chinese Medicine, Harbin, China; ^2^ The Second Affiliated Hospital of Heilongjiang University of Chinese Medicine, Harbin, China

**Keywords:** *Acori tatarinowii rhizoma*, α-asarone, β-asarone, dementia, cognitive functions

## Abstract

**Objective:**

This study aims to elucidate the mitigating effects of the volatile oil of *Acori tatarinowii rhizoma* (ATR) on dementia, in order to provide a reference for future research and applications of the volatile oil of ATR in the field of dementia.

**Materials and methods:**

A search strategy was developed using terms such as “Acori tatarinowii rhizoma,” “Acorus tatarinowii Schott,” “Asarone,” and “Dementia.” The literature search was conducted in PubMed, Web of Science, and Google Scholar, and studies not meeting the inclusion criteria were excluded. This study summarizes the main metabolites, active ingredients, toxicological properties, and pharmacokinetic characteristics of the volatile oil from ATR in mitigating dementia, with a particular focus on its potential mechanisms of action. Furthermore, the study highlights the limitations of existing research and offers insights into future research directions.

**Results:**

The volatile oil of ATR mitigates dementia through multiple pathways, including reducing abnormal protein aggregation, promoting neurogenesis, inhibiting neuronal apoptosis, regulating neurotransmitters, improving synaptic function, modulating autophagy, countering cellular stress, reducing neuroinflammation, and alleviating vascular risk factors.

**Conclusion:**

The multi-pathway pharmacological effects of the volatile oil of ATR are well-aligned with the complex mechanisms of dementia progression, highlighting its significant therapeutic potential for anti-dementia applications. This provides new perspectives for the development of more effective anti-dementia drugs. Nonetheless, further rigorous and high-quality preclinical and clinical investigations are required to address key issues, including the chemical characterization of the volatile oil of ATR, potential synergistic effects among active ingredients, toxicity profiles, and definitive clinical efficacy.

## 1 Introduction

Dementia is a clinical syndrome primarily characterized by progressive cognitive decline. As cognition deteriorates, most individuals with dementia develop psychological and behavioral abnormalities, and may even lose the ability to live independently, imposing substantial physical, mental, and economic burdens on both patients and caregivers (Wang C. et al., 2022). Data indicate that dementia caused a global economic loss of $1.3 trillion in 2019, with global dementia cases surpassing 55 million and increasing at a rate of 10 million new cases every year (WHO, 2023). By 2050, the worldwide number of dementia cases is projected to reach 150 million (Nichols et al., 2022), which will lead to an even greater burden of disease. Despite the already alarming statistics, the destructive impact of dementia may still be underestimated, as it is often misconceived in certain developing countries as an inevitable consequence of aging and, consequently, overlooked (Alladi and Hachinski, 2018). Undoubtedly, dementia has become one of the greatest public health challenges globally.

Based on pathological characteristics, dementia can be classified into several subtypes, including Alzheimer’s disease (AD), vascular dementia (VD), dementia with Lewy bodies (DLB), Frontotemporal dementia (FTD), Parkinson’s disease dementia (PD), and mixed dementias (MD). Numerous hypotheses have been proposed to explain the mechanisms underlying dementia onset and progression, such as the amyloid-beta (Aβ) hypothesis, the microtubule-associated protein tau (Tau) hyperphosphorylation hypothesis, mitochondrial dysfunction theory, neuroinflammation hypothesis, and the cholinergic hypothesis. Current drugs are often developed based on these theories, examples of which include cholinesterase inhibitors, memantine, and Aβ-targeting monoclonal antibodies. Unfortunately, none of these theories appear to independently provide a comprehensive explanation for the pathogenesis of dementia. For instance, the removal of insoluble Aβ plaques has not achieved the anticipated therapeutic effects in alleviating AD symptoms (Li and Selkoe, 2020). Consequently, the limited efficacy of drugs developed solely on the basis of a single hypothesis is unsurprising. Although each dementia subtype exhibits specific pathological characteristics, their boundaries are not always clear, and overlapping features may exist. With ongoing research, increasing evidence suggests that dementia may result from the interplay of multiple pathological mechanisms (Khan et al., 2016), highlighting the urgent global need for more comprehensive prevention and treatment strategies.

An increasing number of studies are exploring the use of medicinal plants or their extracts for mitigating dementia. These natural products may serve as a valuable resource for advancing the development of anti-dementia drugs. *Acori tatarinowii rhizoma* (ATR), the dried rhizome of *Acorus tatarinowii Schott* from the family Araceae, is a traditional medicine widely used in Asian countries such as China, Japan, Korea, and India for conditions such as stroke, dementia, epilepsy, insomnia, and gastralgia (Lam et al., 2016a). Research has shown that ATR and its extracts exhibit neuroprotective, anti-dementia, and antidepressant effects (Kim C. J. et al., 2022). Certain traditional medicine formulations containing ATR, such as Kaixinsan (Fu et al., 2019), Qisheng Wan formula (Xiong et al., 2022), and Sagacious Confucius’ Pillow Elixir (Hou et al., 2023), have demonstrated therapeutic effects on cognitive impairment, dementia, and depression. The plant metabolites of ATR include non-volatile metabolites such as alkaloids, flavonoids, and organic acids, as well as volatile metabolites, particularly phenylpropanoids and terpenoids. The volatile metabolites, primarily the volatile oil, are considered the main active ingredients of ATR (Wen et al., 2023). The volatile oil of ATR exhibits antidepressant, anticonvulsant, neuroprotective, and cognition-enhancing properties (Wang et al., 2023). Its main active ingredients can penetrate the blood-brain barrier (BBB) and are widely distributed within the brain (Liu and Fang, 2011; Lu et al., 2014; Balakrishnan et al., 2022; Chellian et al., 2017), forming the basis for its potential efficacy in mitigating dementia.

Given the complex mechanisms underlying dementia and the pharmacological properties of the volatile oil of ATR, this study highlights its potential as a candidate drug for mitigating dementia. Accordingly, the study aimed to elucidate the pharmacological mechanisms of the volatile oil of ATR in mitigating dementia. A comprehensive search strategy was developed using the following terms: “Acori tatarinowii rhizoma,” “Acorus tatarinowii Schott,” “Asarone,” “Dementia,” “Alzheimer’s Disease,” “Vascular Dementia,” “Dementia with Lewy Bodies,” “Frontotemporal Dementia,” “Parkinson’s Disease Dementia,” “cognitive function,” “central nervous system,” “neuroprotection,” “Neuron,” “neurodegeneration,” “pharmacology,” “pharmacokinetics,” and “toxicity.” Searches were conducted in PubMed, Web of Science, and Google Scholar for studies published from the inception of each database through 1 December, 2024. Duplicates, reviews, editorials, abstracts, comments, irrelevant studies, and those that did not meet the ConPhyMP—Guidelines (Heinrich et al., 2022) were excluded by screening titles, abstracts, or full-text reviews, while the remaining studies were included. These studies enhance our understanding of the potential of the volatile oil of ATR in mitigating dementia and provide valuable insights for the development of future anti-dementia drugs.

## 2 Main active ingredients of the volatile oil of ATR

**FIGURE 1:**
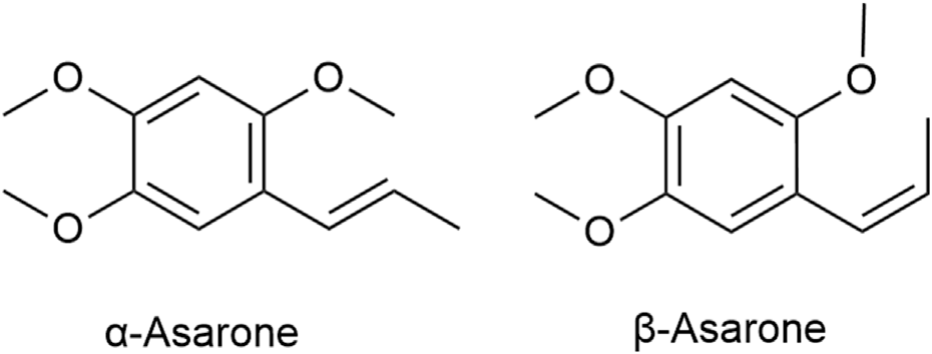
Chemical structure of the main plant metabolites in the volatile oil of ATR.

The volatile oil are the main active ingredients of ATR, serving as crucial indices for its identification and evaluation (Lam et al., 2016b). A variety of metabolites have been identified in the volatile oil of ATR, which can be classified into four main categories: terpenoids, phenylpropanoids, aromatic compounds, and other aliphatic compounds (Yan et al., 2020a). Researchers used analytical testing techniques such as chromatography and GC-MS to analyze the plant metabolites in the volatile oil of ATR derived from different origins and batches (Table 1). While minor variations exist across sources, phenylpropanoids such as α-Asarone and β-Asarone (Figure 1) are consistently the main metabolites of the volatile oil of ATR. These metabolites are not only the characteristic components of the volatile oil of ATR but also the main active ingredients responsible for its dementia-mitigating effects (Wen et al., 2023; Lam et al., 2016b; Lam et al., 2019). Although α-Asarone and β-Asarone are not unique to *A. tatarinowii Schott* and are also present in other plants, such as *Guatteria gaumeri Greenman* (Annonaceae), *Asarum europaeum Linné* (Aristolochiaceae), as well as *Acorus calamus Linn* and *Acorus gramineus Soland* (Acoraceae) (Chellian et al., 2017; Uebel et al., 2021). *Acorus tatarinowii Schott* has emerged as a significant focus in research on dementia mitigation. This prominence is attributable to its high and stable levels of α-Asarone and β-Asarone, its long-standing use in traditional medicine for dementia treatment, and robust support from modern scientific studies (Wen et al., 2023; Wang et al., 2023; Lam et al., 2016b). In contrast, other plants containing these metabolites have limited applications in dementia research or treatment due to their lower metabolite levels, reduced stability, lack of traditional use in dementia therapy, and insufficient preclinical or clinical evidence (Ozarowski, 1956; Lam et al., 2017a; Leclercq et al., 1985; Maseehullah et al., 2022; Hernández et al., 1993; Chamorro et al., 1993). Also based on the aforementioned context, current research on the volatile oil of ATR primarily focuses on its two major active ingredients, while other metabolites are often overlooked due to their lower abundance. Existing studies have demonstrated that the volatile oil of ATR and its main active ingredients exhibit neuroprotective effects through mechanisms involving the reduction of abnormal protein aggregation (Deng et al., 2020), anti-inflammatory activity (Xiao et al., 2019), anti-apoptotic effects (Geng et al., 2010), autophagy-regulating effects (Han et al., 2020), antioxidant effects (Lam et al., 2017b), and regulation of neurotransmitters (Feng et al., 2024). These mechanisms underscore a broad spectrum of actions. This study reviews the pharmacological mechanisms of the volatile oil of ATR and its main active ingredients in the context of dementia mitigation.

**TABLE 1:** The main metabolites in the volatile oil of ATR.

Metabolites	Formula	CAS no.	Extraction method	References
β-Asarone	C_12_H_16_O_3_	5273-86-9	Hydro-distillation	Yan et al. (2020a)
α-Asarone	C_12_H_16_O_3_	2883-98-9	Hydro-distillation	Yan et al. (2020a)
cis-Methyl isoeugenol	C_11_H_14_O_2_	6380-24-1	Hydro-distillation	Yan et al. (2020a)
trans-methylisoeugenol	C_11_H_14_O_2_	6379-72-2	Hydro-distillation	Yan et al. (2020a)
γ-Asarone	C_12_H_16_O_3_	5353-15-1	Hydro-distillation	Yan et al. (2020a)
Methyl eugenol	C_11_H_14_O_2_	93-15-2	Hydro-distillation	Yan et al. (2020a)
Elemicin	C_12_H_16_O_2_	487-11-6	Hydro-distillation	Yan et al. (2020a)
o-Cymene	C_10_H_14_	527-84-4	Hydro-distillation	Yan et al. (2020a)
Estragole	C_10_H_12_O	140-67-0	Hydro-distillation	Yan et al. (2020a)
Aristolone	C_15_H_22_O	25274-27-5	Hydro-distillation	Yan et al. (2020a)
Aihydroagarofuran	C_15_H_26_O	5956-09-2	Hydro-distillation	Yan et al. (2020a)
Isocalamendiol	C_15_H_26_O_2_	25330-21-6	Hydro-distillation	Yan et al. (2020a)
α-Cadinol	C_15_H_26_O	481-34-5	Hydro-distillation	Yan et al. (2020a)
tau-Cadinol	C_15_H_26_O	5937-11-1	Hydro-distillation	Yan et al. (2020a)
Dehydroxy isocalamendiol	C_15_H_24_O	1005276-30-1	Hydro-distillation	Yan et al. (2020a)
Viridiflorol	C_15_H_26_O	552-02-3	Hydro-distillation	Yan et al. (2020a)
Spathulenol	C_15_H_24_O	6750-60-3	Hydro-distillation	Yan et al. (2020a)
Germacrene D-4-ol	C_15_H_26_O	74841-87-5	Hydro-distillation	Yan et al. (2020a)
Eremophila ketone	C_15_H_24_O	158930-41-7	Hydro-distillation	Yan et al. (2020a)
Elemol	C_15_H_26_O	639-99-6	Hydro-distillation	Yan et al. (2020a)
α-Calacorene	C_15_H_20_	21391-99-1	Hydro-distillation	Yan et al. (2020a)
α-Panasinsen	C_15_H_24_	56633-28-4	Hydro-distillation	Yan et al. (2020a)
Isoshyobunone	C_15_H_24_O	21698-46-4	Hydro-distillation	Yan et al. (2020a)
δ-Cadinene	C_15_H_24_	483-76-1	Hydro-distillation	Yan et al. (2020a)
Shyobunone	C_15_H_24_O	21698-44-2	Hydro-distillation	Yan et al. (2020a)
Germacrene D	C_15_H_24_	317819-80-0	Hydro-distillation	Yan et al. (2020a)
γ-Muurolene	C_15_H_24_	30021-74-0	Hydro-distillation	Yan et al. (2020a)
α-Acoradiene	C_15_H_24_	28400-13-7	Hydro-distillation	Yan et al. (2020a)
α-Caryophyllene	C_15_H_24_	6753-98-6	Hydro-distillation	Yan et al. (2020a)
Calarene	C_15_H_24_	17334-55-3	Hydro-distillation	Yan et al. (2020a)
β-Caryophyllene	C_15_H_24_	87-44-5	Hydro-distillation	Yan et al. (2020a)
Longifolene	C_15_H_24_	475-20-7	Hydro-distillation	Yan et al. (2020a)
β-Elemene	C_15_H_24_	515-13-9	Hydro-distillation	Yan et al. (2020a)
α-Patchoulene	C_15_H_24_	560-32-7	Hydro-distillation	Yan et al. (2020a)
Longicyclene	C_15_H_24_	1137-12-8	Hydro-distillation	Yan et al. (2020a)
α-Longipinene	C_15_H_24_	5989-08-2	Hydro-distillation	Yan et al. (2020a)
δ-Elemene	C_15_H_24_	20307-84-0	Hydro-distillation	Yan et al. (2020a)
α-Terpineol	C_10_H_18_O	98-55-5	Hydro-distillation	Yan et al. (2020a)
β-Cedrene	C_15_H_24_	546-28-1	Hydro-distillation	Yan et al. (2020a)
Terpine-4-ol	C_10_H_18_O	562-74-3	Hydro-distillation	Yan et al. (2020a)
endo-Borneol	C_10_H_18_O	464-45-9	Hydro-distillation	Yan et al. (2020a)
2-Bornanone	C_10_H_16_O	464-49-3	Hydro-distillation	Yan et al. (2020a)
Linalool	C_10_H_18_O	78-70-6	Hydro-distillation	Yan et al. (2020a)
γ-Terpinene	C_10_H_16_	99-85-4	Hydro-distillation	Yan et al. (2020a)
Eucalyptol	C_10_H_18_O	470-82-6	Hydro-distillation	Yan et al. (2020a)
d-Limonene	C_10_H_16_	138-86-3	Hydro-distillation	Yan et al. (2020a)
α-Terpinene	C_10_H_16_	99-86-5	Hydro-distillation	Yan et al. (2020a)
β-Pinene	C_10_H_16_	127-91-3	Hydro-distillation	Yan et al. (2020a)
Camphene	C_10_H_16_	79-92-5	Hydro-distillation	Yan et al. (2020a)
α-Pinene	C_10_H_16_	2437-95-8	Hydro-distillation	Yan et al. (2020a)
Caryophyllene oxide	C_15_H_24_O	1139-30-6	Hydro-distillation	Yan et al. (2020a)
Limonene	C_10_H_16_	138-86-3	Hydro-distillation	Yan et al. (2020a)
Benzaldehyde	C_7_H_6_O	100-52-7	Hydro-distillation	Lam et al. (2017a)
L-Linalool	C_10_H_18_O	126-91-0	Hydro-distillation	Lam et al. (2017a)
Camphor	C_10_H_16_O	76-22-2	Hydro-distillation	Lam et al. (2017a)
L-Borneol	C_10_H_18_O	464-45-9	Hydro-distillation	Lam et al. (2017a)
Longipinene	C_15_H_24_	5989-08-2	Hydro-distillation	Lam et al. (2017a)
Shyobunone	C_15_H_24_O	21698-44-2	Hydro-distillation	Lam et al. (2017a)
β-Cadinene	C_15_H_24_	523-47-7	Hydro-distillation	Lam et al. (2017a)
Elemicine	C_12_H_16_O_3_	487-11-6	Hydro-distillation	Lam et al. (2017a)
Patchoulene	C_15_H_24_	514-51-2	Hydro-distillation	Lam et al. (2017a)
Isoelemicine	C_12_H_16_O_3_	5273-85-8	Hydro-distillation	Lam et al. (2017a)
γ-Cadinene	C_15_H_24_	39029-41-9	Hydro-distillation	Lam et al. (2017a)
α-Humulene	C_15_H_24_	6753-98-6	Hydro-distillation	Lam et al. (2017a)
Benzyl benzoate	C_14_H_12_O_2_	120-51-4	Hydro-distillation	Lam et al. (2017a)
Diacetone alcohol	C_6_H_12_O_2_	123-42-2	Hydro-distillation	Lam et al. (2017a)
Bornyl acetate	C_12_H_20_O_2_	76-49-3	Hydro-distillation	Lam et al. (2017a)

## 3 Pharmacokinetics of the volatile oil of ATR

α-Asarone and β-Asarone, both classified as phenylpropanoids, have structural similarities and exhibit similar pharmacokinetic behavior. After oral administration of the volatile oil of ATR in Wistar rats, the peak plasma concentration (T_max_) of α-Asarone is 1.58 ± 0.19 h, with a half-life (T1/2) of 2.93 ± 0.67 h, while β-Asarone reaches its T_max_ at 1.42 ± 0.18 h, with a T1/2 of 2.12 ± 0.22 h (Wang et al., 2014). Direct oral administration of α-Asarone and β-Asarone results in faster absorption and distribution. Studies in Sprague-Dawley (SD) rats showed that after administering an α-Asarone solution orally, T_max_ reached 11.67 ± 4.08 min, and the terminal T1/2 was 66.99 ± 29.76 min (Qian et al., 2015). For β-Asarone, oral administration resulted in a T_max_ of 12 min and a terminal T1/2 of 54 min (Wu and Fang, 2004). Both α-Asarone and β-Asarone can be quickly absorbed and distributed, but more importantly, they can penetrate the BBB and act on brain regions such as the hypothalamus, hippocampus, brainstem, cortex, and cerebellum (Liu and Fang, 2011; Lu et al., 2014; Balakrishnan et al., 2022; Chellian et al., 2017), forming the pharmacological basis for their dementia mitigation effects. It must be noted, however, that while many studies suggest that ATR extracts or their main active ingredients can cross the BBB and alleviate BBB dysfunction (Li et al., 2025; Gao X. et al., 2022; Luo et al., 2025), some studies also imply that the ability of ATR extracts or their main active ingredients to penetrate the BBB may be associated with an increase in BBB permeability (Wu et al., 2018; Hu et al., 2009). This could potentially raise the likelihood of toxic substances entering the brain, which may pose risks for dementia patients. Such discrepancies highlight the need for further in-depth research into the mechanisms by which the volatile oil of ATR or its main ingredients cross the BBB, in order to determine whether they have potential side effects.

The high lipophilicity of α-Asarone and β-Asarone facilitates their crossing of the BBB, however, it also leads to poor solubility in water, resulting in low oral bioavailability. The bioavailability of α-Asarone tablets in humans is reportedly less than 10% (Chellian et al., 2017; Deng et al., 2017). Research has suggested that intranasal delivery and intravenous injection can significantly enhance both absolute bioavailability and maximum plasma drug concentration. However, solubility promoters in α-Asarone injection solutions have been associated with severe allergic reactions (Deng et al., 2017). Intranasal delivery appears to be a better option, as part of the drug can directly target brain tissues via the nasal cavity, bypassing first-pass metabolism in the liver, thus improving bioavailability, maximum plasma concentration, and drug retention time in the body. Intranasal delivery also reduces high systemic concentrations, minimizing toxic side effects in blood and liver (Lu et al., 2014; Pan et al., 2018).

## 4 Toxicological studies of the volatile oil of ATR

While ATR is traditionally considered safe for medicinal use, toxicological studies on the volatile oil of ATR and its active ingredients remain necessary and warrant further investigation. Current toxicological research on the volatile oil and its main active ingredients is limited, and results are inconsistent.

In acute toxicity studies, oral administration of up to 5,000 mg/kg of ATR aqueous extract (containing volatile oil) in mice, a dosage far exceeding traditional uses, caused no toxicity or fatalities. In subacute experiments, daily doses of 1,250, 2,500, and 5,000 mg/kg over 28 days also resulted in no toxicity or fatalities, though some mild renal damage was observed in the high-dose group (5,000 mg/kg) (Liu et al., 2024). These findings suggest a high safety margin for clinical use, particularly for short-term, moderate doses, but long-term, high-dose use may carry potential nephrotoxic risks.

Toxicological studies on α-Asarone and β-Asarone are relatively more extensive. Acute toxicity experiments revealed that the median lethal dose (LD50) of oral α-Asarone in mice exceeds 1,000 mg/kg (Chen et al., 2013), while the LD50 for intraperitoneal injection is 245.2 mg/kg (Morales-Ramírez et al., 1992). For β-Asarone, the oral LD50 in rats is 1,010 mg/kg (Food, 2002), and the intravenous LD50 in mice is 1,560 mg/kg (Liu et al., 2013). In subacute toxicity experiments, mice receiving daily oral α-Asarone at doses of 50, 100, and 200 mg/kg for 28 days showed no significant adverse effects, although spontaneous activity decreased in the high-dose group (200 mg/kg) (Chen et al., 2013). Similarly, intraperitoneal injection of 100 mg/kg β-Asarone for five consecutive days in juvenile SD rats caused no toxicity or fatalities (Food, 2002). In chronic toxicity studies, intraperitoneal administration of α-Asarone (≤156 mg/kg) or β-Asarone (≤52 mg/kg) in juvenile mice increased the incidence of hepatocellular adenomas and carcinomas at 10–13 months compared to controls (KIM et al., 1999; Wiseman et al., 1987). However, a 30-day course of intraperitoneal injections of α-Asarone (9 mg/kg) in rats did not cause liver morphological changes (Manikandan and Devi, 2005). Oral administration of β-Asarone (200 μg/kg) in mice for 20 weeks caused no toxicity, while daily oral doses of 10, 20, or 50 mg/kg for 90 days caused abnormal blood parameters only at the highest dose (50 mg/kg) (Liu et al., 2013).

Certain bacterial reverse mutation assays showed that α-Asarone and β-Asarone exhibited no mutagenicity in *Salmonella typhimurium* strains (TA98, TA102, TA1535, TA1537, TA1538), regardless of metabolic activation. However, mutagenicity was observed in TA100 with metabolic activation (Marczewska et al., 2013; Berg et al., 2016; Cassani-Galindo et al., 2005; Göggelmann and Schimmer, 1983). Additionally, while 3′-hydroxylated metabolites of α-Asarone and β-Asarone showed no mutagenicity in human sulphotransferase-expressing *Salmonella* strains, β-Asarone’s side-chain epoxide metabolite demonstrated mutagenicity (Berg et al., 2016). Embryotoxicity studies revealed no teratogenic effects of α-Asarone in pregnant rats (Jimenez et al., 1988). However, in mice, α-Asarone showed embryotoxicity (15–60 mg/kg) and maternal toxicity (60 mg/kg), with potential teratogenic effects (Salazar et al., 1992). Short-term exposure to α-Asarone caused reproductive toxicity, such as reduced sperm concentration, motility, and seminal vesicle weight in CF1 mice at high doses (30 mg/kg intraperitoneally for 5 days) (Chamorro et al., 1998). However, long-term subacute toxicity studies (10 and 20 mg/kg, orally, 5 days per week for eight consecutive weeks) showed no significant effects on sperm count, testicular tissue morphology, testicular weight, or epididymal weight (Chamorro et al., 1999). Debates persist regarding the genotoxicity of α-Asarone and β-Asarone. Oral administration of α-Asarone at a dosage of 50 mg/kg does not influence the formation of micronuclei in the peripheral blood reticulocytes of normal mice (Sandeep and Nair, 2011), and injection of β-Asarone at a dosage of 182.4 mg/kg also shows no effect on the formation of micronuclei in polychromatic erythrocytes within the mouse bone marrow (Ashadevi et al., 2014). These findings suggest that the tested doses of α-Asarone and β-Asarone do not possess significant genotoxic effects. However, α-Asarone caused DNA damage in human lymphocytes (Morales-Ramírez et al., 1992), rat hepatocytes (Hasheminejad and Caldwell, 1994), and L929 fibroblast cells (Marczewska et al., 2013), indicating genotoxicity, while β-Asarone exhibited genotoxicity in *Escherichia coli* (Kevekordes et al., 1999), rat hepatocytes (Hasheminejad and Caldwell, 1994), hamster pulmonary fibroblasts (Haupenthal et al., 2015), HepG-2 cells, and human lymphocytes (Kevekordes et al., 2001).

In summary, while existing toxicity endpoints suggest a sufficient safety margin for regular therapeutic use of the volatile oil of ATR and its main active ingredients, more comprehensive and systematic toxicological studies are needed. These studies should conform to internationally recognized testing guidelines, such as OECD test guidelines, to better evaluate the safety profile of these plant metabolites.

## 5 Pharmacological effects of the volatile oil of ATR in mitigating dementia

**FIGURE 2:**
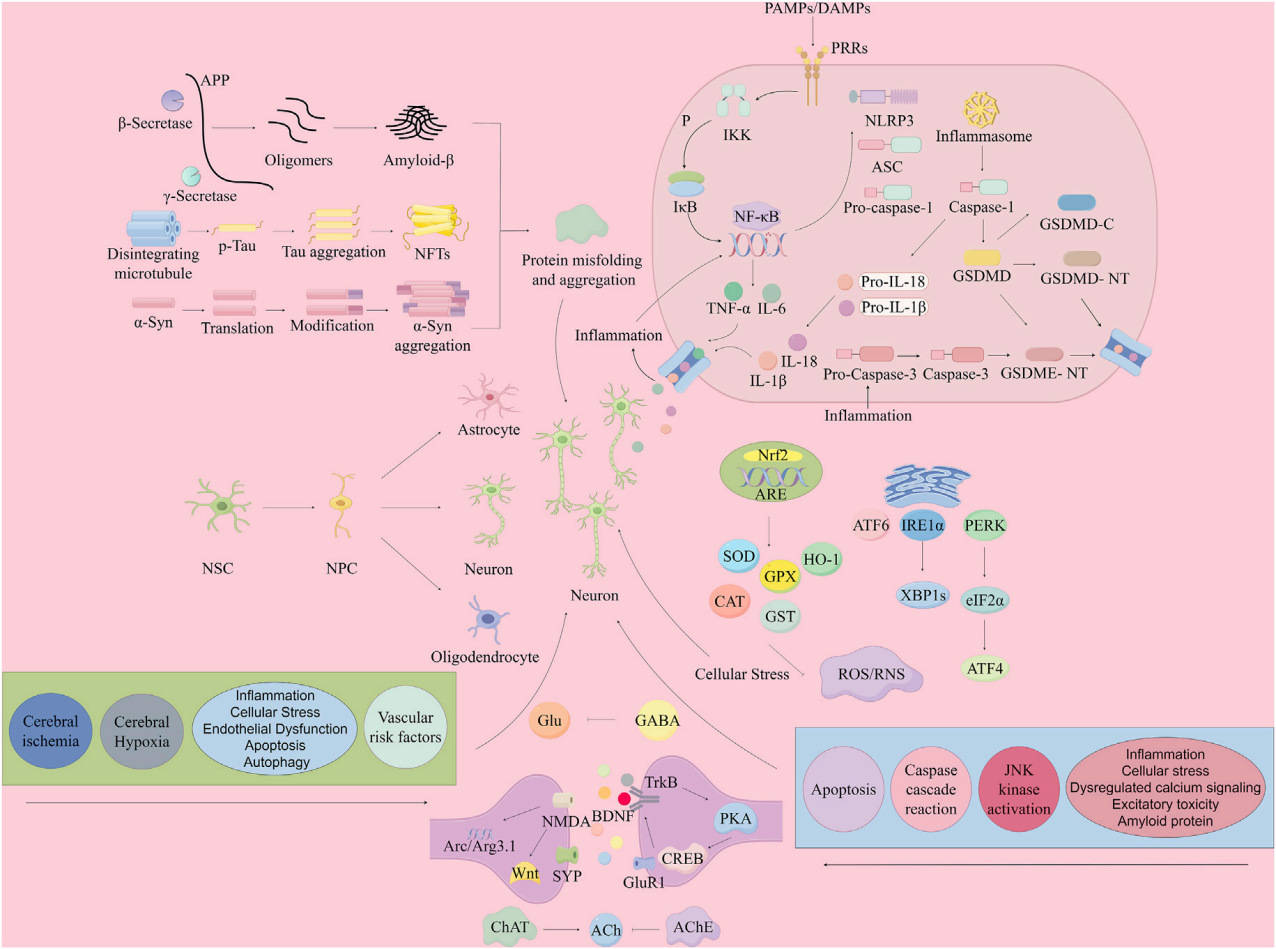
Mechanisms of neuronal damage in dementia (mitigation by the volatile oil of ATR). By Figdraw. Note: APP, Amyloid Precursor Protein; Aβ, Amyloid-β; NFTs, Neurofibrillary Tangles; NSC, Neural Stem Cells; NPC, Neural Progenitor Cells; PAMPs, Pathogen-Associated Molecular Patterns; DAMPs, Damage-Associated Molecular Patterns; PRRs, Pattern Recognition Receptors; IKK, Inhibitor of Kappa light polypeptide gene enhancer in B-cells Kinase; NF-κB, Nuclear Factor kappa-light-chain-enhancer of activated B cells; TNF-α, Tumor Necrosis Factor-alpha; IL-1β, Interleukin 1 beta; IL-18, Interleukin 18; IL-6, Interleukin 6; ASC, Apoptosis-associated speck-like protein containing a CARD; NLRP3, Nucleotide-binding oligomerization domain-like receptor family pyrin domain containing 3; GSDMD, Gasdermin D; GSDME, Gasdermin E; GSDMD-C, Gasdermin D C-terminal; GSDMD-NT, Gasdermin D N-terminal; GSDME-NT, Gasdermin E N-terminal; Caspase, Cysteinyl aspartate specific protease; Caspase-1, Cysteinyl aspartate specific protease 1; Caspase-3, Cysteinyl aspartate specific protease 3; PERK, Protein Kinase R-like endoplasmic reticulum kinase; ATF, Activating Transcription Factor; XBP1s, X-box binding protein 1 splicing; ROS/RNS, Reactive Oxygen Species/Reactive Nitrogen Species; SOD, Superoxide Dismutase; CAT, Catalase; CREB, cAMP Response Element-binding Protein; BDNF, Brain-Derived Neurotrophic Factor; ACh, Acetylcholine; NMDA, N-methyl-D-aspartate; PKA, Protein Kinase A; CaMKII, Calcium/calmodulin-dependent protein kinase II; ChAT, Choline Acetyltransferase.

Current evidence indicates that the volatile oil of ATR effectively mitigate dementia. Research primarily focuses on α-Asarone and β-Asarone, examining their intervention in the pathogenesis of dementia. Key mechanisms include reducing abnormal protein aggregation, promoting neurogenesis, inhibiting neuronal apoptosis, modulating neurotransmitters, improving synaptic function, regulating autophagy, mitigating cellular stress, inhibiting neuroinflammation, and mitigating vascular risk factors (Table 2; Figure 2).

**TABLE 2:** Pharmacological effects of the volatile oil of ATR in mitigating dementia.

Active ingredient	Method	Dose	Model	Targets	Action	References
α-Asarone	*In vitro/In vivo*	*In vivo*: 10, 25 mg/kg *In vitro*: 2.5, 7.5 μg/mL	SD rats, primary hippocampal neurons of SD rats, HEK293 cells	Aβ_1-42,_ Cell viability, GABA_A_R, calcium level	Reducing abnormal protein aggregation	Chen et al. (2020a)
	*In vivo*	7.5, 15, 30 mg/kg	C57BL/6 J mice	Glu, GABA, GluR2, NMDAR2B, pSYNΙ, GLT-1, CaMKⅡ	Modulating neurotransmitters	Li et al. (2019)
	*In vitro*	15, 30, 50 μM	Astrocytes from SD rats	BDNF, NGF, GDNF, PKA, ERK, CREB	Increasing neurotrophic factor levels	Lam et al. (2019)
	*In vitro*	0, 0.3, 1, 3, 10 μM	NPCs from adult mouse hippocampi, primary neurons from postnatal mouse cortex and hippocampus	NPC, Tuj1, Tbr2, Ki67, Sox2, c-Fos, Nestin, GFAP, Neuroblasts, immature neurons, NeuN, GFAP; p-ERK, p-Akt	Promoting neurogenesis	Mao et al. (2015)
	*In vitro*	1, 5, 10 μM	Mouse macrophage-like cell line J774A1	Cell viability, cytochrome C, Apaf-1, caspase-12, caspase-9, caspase-3, IRE1α, XBP1, TRAF2, JNK1/2, ASK1, PERK, ATF4	Mitigating cellular stress, inhibiting neuronal apoptosis, mitigating vascular risk factors	Park et al. (2016)
	*In vivo*	15, 30 mg/kg	Wistar rats	AChE	Modulating neurotransmitters	Venkatesan (2022)
	*In vitro/In vivo*	*In vivo*: 10, 20 mg/kg *In vitro*: 12, 24, 48 μM	SD rats,PC-12 cells	cell viability, apoptosis rate, Neuronal morphology, GFAP, Iba-1, p62, LC3I, LC3II	Inhibiting neuronal apoptosis, regulating autophagy	Zhang et al. (2021a)
	*In vivo*	10 mg/kg	Wistar rats	NO, GFAP, neuronal damage	Mitigating cellular stress	Limón et al. (2009a)
	*In vitro*	0.5, 5, 50 μM	HT22 cells	cell death, cell viability, ROS, PERK, p-PERK, BiP, GRP94	Mitigating cellular stress	Mikami et al. (2021)
	*In vivo*	30, 60 mg/kg	APP/PS1 mice	Aβ42, p-tau, GFAP, GAD, TNF-α, IL-1β, IL-6, Nissl bodies, autophagosomes	Reducing abnormal protein aggregation, inhibiting neuroinflammation, regulating autophagy	Zeng et al. (2021)
β-Asarone	*In vivo*	10, 20, 40 mg/kg	APP/PS1 mice	Aβ, Aβ_40_, Aβ_42_, APP	Reducing abnormal protein aggregation	Deng et al. (2020)
	*In vitro/In vivo*	*In vivo*: 10 mg/kg *In vitro*: 100 μM	C57BL/6 mice; SH-SY5Y cells	MALAT1, α-synuclein, cell viability	Reducing abnormal protein aggregation	Zhang et al. (2016a)
	*In vitro*	0, 1, 10, 100, 1,000 μM	PC-12 cells	Aβ_1-40_, Ca^2+^	Protecting neuron	Irie and Keung (2003)
	*In vitro*	15, 30, 50 μM	Astrocytes from SD rats	BDNF, NGF, GDNF, PKA, ERK, CREB	Increasing neurotrophic factor levels	Lam et al. (2019)
	*In vitro*	0, 0.3, 1, 3, 10 μM	NPCs from adult mouse hippocampi, primary neurons from postnatal mouse cortex and hippocampus	NPC, Tuj1, Tbr2, Ki67, Sox2, c-Fos, Nestin, GFAP, Neuroblasts, immature neurons, NeuN, GFAP; p-ERK, p-Akt	Promoting neurogenesis	Mao et al. (2015)
	*In vitro*	10 μg/mL (pretreatment) 0.01, 0.1, 1, 10, 100, 1000 μg/mL	SH-SY5Y cells	Cell proliferation, LDH, Apoptosis, ASK1, MKK7, JNK, Bax, Bad, cytochrome C, caspase-9, caspase-3	Inhibiting neuronal apoptosis	Zou et al. (2011)
	*In vivo*	12.5, 25, 50 mg/kg	SD rats	Cell proliferation, Bax, Bad, Caspase 9, ASK1, MKK7, c-Jun	Inhibiting neuronal apoptosis	Liu et al. (2010)
	*In vitro/In vivo*	*In vivo*: 7, 21 mg/kg *In vitro*: 0, 1, 3, 30, 100, 300 μg/mL	APP/PS1 mice, PC-12 cells, cortical neurons	Cell viability, apoptotic cells, CaMKII-α, CREB, p-CREB, Bcl-2, Caspase-3	Inhibiting neuronal apoptosis	Wei et al. (2013)
	*In vitro*	12, 24, 36, 72, 144 μM	PC-12 cells	LC3, BECN1, PINK1, Parkin, APP, PS1, Aβ, SYN1	Regulating autophagy	Wang et al. (2019)
	*In vivo*	7.5, 15, 30 mg/kg	SD rats	α-syn, PI3K, p-PI3K, Akt, p-Akt, mTOR, p-mTOR, Beclin-1, p62	Regulating autophagy	Wang et al. (2024)
	*In vitro/In vivo*	*In vivo*: 10, 20, 40 mg/kg *In vitro*: 10, 50, 100 μM	SD rats, SN4741 cells	HVA, 5-HIAA, DOPAC, 5-HT, TH, α-syn, LC3-II, p62, Beclin-1, Bcl-2, JNK	Modulating neurotransmitters, regulating autophagy	Zhang et al. (2016b)
	*In vitro/In vivo*	*In vivo*: 21.2, 42.4, 84.8 mg/kg *In vitro*: 6.25, 12.5, 25 μM	APP/PS1 mice, NG108-15 cells	SYP, GluR1, cell morphology, Cell proliferation	Improving synaptic function	Liu et al. (2016)
	*In vivo*	12.5, 25, 50 mg/kg	Wistar rats	SOD, GPX	Mitigating cellular stress	Saki et al. (2020)
	*In vitro*	0, 10, 30, 60, 120 μM	PC12 cells	Cel viability, Bcl-2, Bax, caspase-3, MDA, SOD, CAT, GSH-PX, ROS, Nrf2, HO-1, PI3K, Akt	Inhibiting neuronal apoptosis, mitigating cellular stress	Meng et al. (2021)
	*In vitro*	10, 50, 100 μM	SH-SY5Y cells	Cell viability, LC3, Beclin-1, Bcl-2, IL-6, IL-1β, TNF-α	Inhibiting neuronal apoptosis, inhibiting neuroinflammation	Chang and Teng (2015)
	*In vivo*	25, 50, 100 mg/kg	Wistar rats	rCBF, lactic acid, pyruvic acid, Na + K + ATPase, ET-1, eNOS, APP	Mitigating vascular risk factors	Li et al. (2012)
Ethanol extracts of ATR	*In vivo*	10 g/kg	C57BL/6 mice; aged mice; APP/PS1 mice	NPC, Tuj1, Tbr2, Ki67, Sox2, c-Fos, Nestin, GFAP, Neuroblasts, immature neurons, NeuN, GFAP; p-ERK, p-Akt	Promoting neurogenesis	Mao et al. (2015)
Aqueous extracts of ATR	*In vivo*	4, 8, 16 g/kg	3×Tg-AD mice	Tau, p-Tau, myelin injury	Reducing abnormal protein aggregation, alleviating myelin damage	Fu et al. (2020)
Essential oil of Acorus Tatarinowii Schott (by supercritical CO_2_ fluid)	*In vivo*	50, 100 mg/kg	3 × Tg-AD mice	Aβ, Tau, GSK-3β, NeuN, Bax, Bcl-2, Iba-1, GFAP, IL-1β, TNF-α, IL-6, IL-18, cleaved-IL-1β, IKKβ, NF-κB, NLRP3, ASC, Caspase-1, cleaved-Caspase-1, GSDMD-N	Reducing abnormal protein aggregation, inhibiting neuroinflammation, inhibiting neuronal apoptosis	Xu et al. (2023)

Note: Aβ, amyloid-beta; Glu, Glutamate; GABA, γ-aminobutyric acid; NMDA, N-methyl-D-aspartic acid; GluR2, Glutamate Receptor 2; NMDAR2B, N-Methyl-D-Aspartate Receptor 2B; pSYNΙ, phosphorylated synaptophysinⅠ; GLT-1, Glutamate Transporter 1; CaMKII, Calmodulin-dependent protein kinase II; BDNF, Brain-Derived Neurotrophic Factor; NGF, nerve growth factor; GDNF, Glial Cell Line-Derived Neurotrophic Factor; PKA, Protein Kinase A; ERK, Extracellular signal-regulated kinase; CREB, cAMP response element binding; NPC, neural progenitor cell; Tuj1, β-Tubulin class III; Nestin, Neuronal Intermediate Filament Protein; Tbr2, T-box brain gene 2; Ki67, Antigen identified by monoclonal antibody Ki-67; Sox2, SRY-Box Transcription Factor 2; c-Fos, Proto-oncogene c-Fos; Perk, PKR-like ER kinase; GFAP, glial fibrillary acidic protein; NeuN, neuronal nuclei; AChE, acetylcholinesterase; LDH, lactate dehydrogenase; IRE1α, Inositol-Requiring Transcription Factor 1α; XBP1, X-Box Binding Protein 1; TRAF2, TNF, Receptor-Associated Factor 2; JNK1/2, c-Jun N-terminal Kinases 1/2; ASK1, Apoptosis Signal-Regulating Kinase 1; ATF4, Activating Transcription Factor 4; NO, nitric oxide; ROS, reactive oxygen species; BiP, binding immunoglobulin protein; GRP94, Glucose-Regulated Protein 78; GAD, glutamic acid decarboxylase; TNF-α, Tumor Necrosis Factor-alpha; IL-1β, Interleukin-1 beta; IL-6, Interleukin-6; APP, amyloid precursor protein; PS1, Presenilin 1; MALAT1, Metastasis-Associated Lung Adenocarcinoma Transcript 1; α-synuclein, Alpha-synuclein; PI3K, Phosphoinositol-3 kinase; Akt, Protein Kinase B; mTOR, mammalian target of rapamycin; Beclin-1, Bcl-2-Interacting Myosin-Like Coiled-Coil Protein; HVA, homovanillic acid; 5-HT, 5-Hydroxytryptamine; TH, tyrosine hydroxylase; LC3, Microtubule-Associated Protein 1A/1B Light Chain 3; BECN1, Beclin 1; PINK1, PTEN, Induced Putative Kinase 1; SYP, synaptophysin; SOD, superoxide dismutase; GPX, glutathione peroxidase; CAT, catalase; GSH-PX, glutathione peroxidase; Nrf2, Nuclear factor erythroid 2-related factor 2; HO-1, Heme Oxygenase 1; eNOS, endothelial nitric oxide synthase; ET-1, Endothelin-1; rCBF, regional cerebral blood flow; Na+K+ATPase, Sodium-Potassium ATPase; Dcx, Doublecortin; p-ERK, Phospho-Extracellular signal-regulated kinase; p-Akt, Phospho-Protein Kinase B; p-Tau, Phospho-Tau; GSK-3β, Glycogen Synthase Kinase-3 beta; Bax, Bcl-2 Associated X Protein; Bcl-2, B-cell lymphoma 2; Iba-1, Ionized calcium binding adapter molecule 1; IL-18, Interleukin-18; cleaved-IL-1β, Cleaved Interleukin-1 beta; IKKβ, Inhibitor of Nuclear Factor kappa-B kinase subunit beta; NF-κB, Nuclear Factor kappa-light-chain-enhancer of activated B cells; NLRP3, NLR Family Pyrin Domain Containing 3; ASC, Apoptosis-associated speck-like protein containing a CARD; Caspase-1, Cysteinyl Protease Caspase-1; cleaved-Caspase-1, Cleaved Cysteinyl Protease Caspase-1; GSDMD-N, Gasdermin D N-terminus.

### 5.1 Alleviating learning and memory impairments

Learning and memory impairments are pervasive throughout the progression of dementia, with mild cognitive impairment (MCI) serving as a precursor and risk state for dementia (Breton et al., 2019; Gauthier et al., 2006). MCI is characterized by declines in memory, attention, and cognitive abilities beyond what is expected for age and education level (Eshkoor et al., 2015). As a transitional phase, MCI offers a critical window for early intervention to reduce the risk of dementia onset. Learning and memory impairments are associated with pathological factors such as Aβ deposition, tau hyperphosphorylation, neurotransmitter imbalances, excitotoxicity, neuroinflammation, and aberrant neurotrophic signaling. Addressing these impairments is thus of significant importance for preventing and treating dementia.

Studies conducted in elderly rats with cognitive deficits have demonstrated the ability of α-Asarone to lower hippocampal Aβ_1-42_ levels, restore balance between the glutamatergic and GABAergic systems, alleviate excitotoxicity, and improve spatial learning and memory in the Morris water maze (Chen Y. et al., 2020). Similarly, in ethanol-induced learning and memory impairment models in mice, α-Asarone corrected the imbalance of glutamatergic and GABAergic systems and improved the object recognition index in a novel object recognition task (Li et al., 2019). In 3-month-old APP/PS1 transgenic mice, α-Asarone significantly ameliorated learning and memory deficits by reducing Aβ_42_, p-tau, and inflammatory factor levels (Zeng et al., 2021). β-Asarone has shown a potent neuroprotective effect in corticosterone-induced learning and memory impairment models by upregulating BDNF, CREB, and Bcl-2 expression while downregulating Bax, thereby improving cognitive deficits through neurotrophic support and inhibition of neuronal apoptosis (Lee et al., 2015). Synaptic function, a critical determinant of neuronal connectivity strength, plays an essential role in learning and memory (Khaspekov and Frumkina, 2023). β-Asarone promotes synaptic plasticity and synaptogenesis in lead-exposed rats, mitigating learning and memory impairments (Yang QQ. et al., 2016). Additionally, α-Asarone and β-Asarone have been shown to promote hippocampal neurogenesis in both healthy and impaired models, with neurogenesis closely linked to learning and memory enhancement (Mao et al., 2015). These findings highlight the potential of α-Asarone and β-Asarone to improve learning and memory during the MCI phase, thereby reducing the risk of dementia. Even in dementia stages, the volatile oil of ATR have been shown to alleviate such impairments. For instance, aqueous extracts of ATR decreased p-Tau aggregation and repaired myelin damage in the hippocampus of AD mice, significantly improving cognitive abilities (Fu et al., 2020), while β-Asarone improved synaptic plasticity and alleviated cognitive deficits in AD mice (Liu et al., 2016). These findings, across various animal models, collectively demonstrate that the volatile oil of ATR utilize multifaceted mechanisms to enhance learning and memory, underscoring their therapeutic potential for dementia.

### 5.2 Reducing abnormal protein aggregation

The Aβ deposition hypothesis is widely recognized as a central pathological mechanism in dementia. Aβ not only serves as a hallmark of AD but also plays a role in the pathogenesis of other dementia subtypes, such as VD, PD, and DLB (Sengupta and Kayed, 2022). Aβ is generated by sequential proteolysis of amyloid precursor protein (APP) by β-secretase and γ-secretase, resulting in 39–43 amino acid-long peptides, with Aβ_1-40_ and Aβ_1-42_ being the primary subtypes (Schwartz and Ziv, 2008; Ziv and Schwartz, 2008). The production of Aβ is related to APP, β-secretase and γ-secretase (Yang G. et al., 2021), and Aβ clearance occurs through enzymatic degradation, glial cell activity, or autophagy (Cai et al., 2023; Leissring, 2016). Dysfunction in the balance of Aβ production and clearance leads to its accumulation (CAI et al., 2023), transitioning from a soluble monomeric state to insoluble amyloid fibrils (Mattson, 2004; Orgogozo et al., 2003). Aβ deposition causes oxidative stress, damages neuronal cells (Koenig and Meyerhoff, 2003), activates glial cells to trigger inflammation (PHILLIPS et al., 2014; Franco-Bocanegra et al., 2019), and promotes tau hyperphosphorylation (Canudas et al., 2005). Tau is a microtubule-associated protein, mainly found in neurons of the central nervous system, and its main function is to bind to tubulin, promote the assembly and stability of microtubules, and thus maintain the structure and function of the cytoskeleton (Kosik, 1990). Once hyperphosphorylated, tau loses its ability to bind microtubules for cytoskeletal stability, forming intracellular neurofibrillary tangles (NFTs), which disrupt neuronal structure and function and contribute to the pathogenesis of AD, PD, VD, and other dementia subtypes (Sun et al., 2021; Li C. et al., 2024; Pan et al., 2022). α-Synuclein, a presynaptic neuronal protein, is highly enriched in presynaptic nerve endings and is associated with a variety of neurodegenerative diseases (Bennett, 2005). Abnormal aggregation of α-Synuclein is believed to lead to neuronal death, and is also a pathological marker of PD and DLB (Calabresi et al., 2023).

Aqueous extracts of ATR reduced p-Tau aggregation and repaired myelin damage in 3×Tg-AD mice, thereby improving cognitive function (Fu et al., 2020). Myelin sheath is a multilayer lipid and protein composite membrane structure wrapped outside the axon of neurons (Stassart et al., 2018), which can promote the efficiency of action potential conduction and the integration of neural networks, and is a key element in maintaining cognitive function (Chen X. et al., 2020). Recent studies have found that myelin damage may be a driving factor for Aβ deposition (Depp et al., 2023), so the extracts of ATR may alleviate Aβ deposition by reducing myelin damage. Neuronal hyperexcitability may promote the hydrolysis of APP (Canter et al., 2016). Gamma-Aminobutyric Acid (GABA) is the predominant inhibitory neurotransmitter in the central nervous system. α-Asarone can alleviate neuronal hyperexcitability by modulating GABA receptors, thereby mitigating APP hydrolysis and reducing Aβ formation (Chen Y. et al., 2020). Further studies have shown that α-Asarone can reduce neuroinflammation and restore autophagic flux, thereby decreasing Aβ_1-42_ and p-Tau aggregation in the hippocampus of APP/PS1 mice (Zeng et al., 2021). β-Asarone can reduce levels of Aβ_40_, Aβ_42_, and APP in the hippocampus of APP/PS1 mice, thereby mitigating Aβ deposition and inhibiting excessive autophagy. However, the relationship between the two phenomena requires further investigation (Deng et al., 2020). The receptor of advanced glycation end products (RAGE) is a cell surface receptor that can bind to Aβ. High RAGE expression enhances β-secretase activity, thereby increasing Aβ production. The signaling cascades triggered by their interaction are a critical mechanism by which Aβ contributes to AD (Chen et al., 2007). β-Asarone alleviates Aβ_1-42_ levels and Aβ plaque deposition in the cortex and hippocampus of APP/PS1 mice by reducing RAGE levels (Yang C. et al., 2016). β-Asarone reduces α-Synuclein expression levels in the brain tissue of PD mice, which has also been validated *in vitro*. This effect may be associated with the regulation of the long non-coding RNA MALAT1 (Zhang Q. S. et al., 2016). Studies have also indicated that abnormal calcium mobilization within cells may accelerate Aβ formation (Querfurth et al., 1997; Moreno-Ortega et al., 2015). Both α-Asarone and β-Asarone can inhibit Ca^2+^ uptake in PC-12 cells and reduce the cytotoxic effects of Aβ on these cells (Irie and Keung, 2003).

### 5.3 Promoting neurogenesis

Neurogenesis occurs throughout a human’s lifetime, with adult neurogenesis referring to the production of new neurons that are integrated into existing neural circuits of the adult brain. Decline in neurogenesis is closely associated with learning and memory impairments and is a critical factor in the onset and progression of dementia. Neurogenesis contributes to rejuvenating the nervous system and maintaining its functional integrity (Kim T. A. et al., 2022; Isaev et al., 2019). Neurogenesis relies on neural stem cells (NSCs), which are a class of stem cells capable of self-renewal and multipotent differentiation. NSCs can differentiate into three main cell types in the central nervous system: neurons, astrocytes, and oligodendrocytes. They play roles in neuronal nutrition, promoting neuroregeneration, and immune regulation. When neurons are damaged or degenerate, quiescent NSCs in the body are activated, proliferate, migrate to the damaged area, and differentiate, thereby repairing the injured nervous system (Chen et al., 2018). Slowly self-renewing NSCs produce neural progenitor cells (NPCs), which exhibit a faster cell division cycle and eventually differentiate into neurons or glial cells (Huang and Zhang, 2019; Vasic et al., 2019). Extensive neuronal loss is one of the main pathological features of dementia. Enhancing neurogenesis may help replenish deficient neurons in dementia patients, promote the secretion of neurotrophic factors, improve the microenvironment for neuronal survival, and increase neuronal survival rate, thus serving as a potential strategy for the mitigation of dementia (Roda et al., 2022; Zheng, 2022).

NPCs represent an intermediate stage in NSCs differentiation and possess both self-renewal capacities and multipotent differentiation potentials, making them critical for neurogenesis (Zhao et al., 2008). Reduction in NPCs proliferation and renewal may also be a significant contributor to cognitive impairment (Haughey et al., 2002; Seib et al., 2013). Ethanol extracts of ATR, along with its major active ingredients α-Asarone and β-Asarone, have been shown to enhance NPCs proliferation and neurogenesis in the hippocampi of healthy mice, aged mice, and APP/PS1 transgenic mice. *In vitro* studies further support these findings (Mao et al., 2015). NPCs proliferation is regulated by neurotrophic factors, which are a class of proteins that provide nutritional support to neurons. Adequate levels of neurotrophic factors are beneficial for neurogenesis and play key roles in maintaining nervous system function and facilitating injury repair. Numerous neurodegenerative diseases, including dementia, are closely linked to alterations in the expression of neurotrophic factors and their receptors. For instance, levels of brain-derived neurotrophic factor (BDNF), nerve growth factor (NGF), and glial cell line-derived neurotrophic factor (GDNF) are reduced in AD, FTD, PD, LBD, and VD (Mitra et al., 2021; Biane et al., 2014; Ventriglia et al., 2013). Therefore, increasing neurotrophic factor levels may be an effective strategy to restore neuronal function. α-Asarone and β-Asarone may stimulate the expression and secretion of BDNF, NGF, and GDNF by modulating the PKA/ERK/CREB pathway (Lam et al., 2019), thereby maintaining and repairing nervous system function. Another study indicates that β-Asarone might regulate BDNF levels by modulating ERK pathway activity through MKP-1 expression (Sun et al., 2015).

### 5.4 Inhibiting neuronal apoptosis

Apoptosis is a form of programmed cell death that occurs under physiological conditions. It is a process of active cellular death that plays a vital role in regulating development, cell differentiation, and the renewal of normal cells within the body. In the progression of dementia, apoptosis interacts with various pathogenic hypotheses, such as Aβ deposition-induced neuronal damage, oxidative stress, excitotoxicity, and inflammation (Erekat, 2022). A critical issue is that apoptosis leads to neuronal loss, which is a significant cause of dementia (Liang et al., 2024). Apoptosis is regulated through multiple pathways, and the volatile oil of ATR primarily exert their anti-apoptotic effects by modulating microRNA (miRNA), the c-Jun N-terminal kinase (JNK) pathway, and calcium homeostasis. miRNAs are a class of endogenous, non-coding single-stranded RNA molecules that regulate gene expression by binding to the 3′-untranslated region (3′-UTR) of target mRNAs. Evidence suggests that miRNAs contribute to the development and progression of dementia by regulating synaptic function, inflammatory factors, and oxidative stress (Song and Kim, 2017; Zhou et al., 2017; Zhang et al., 2018). JNK plays a crucial role in apoptosis-related gene activation, expression, and regulation. For instance, JNK signaling regulates the expression of members of the B-cell lymphoma 2 (Bcl-2) family, which are key players in apoptosis (Rajesh and Kanneganti, 2022). Activation of JNK can trigger a caspase cascade, wherein executioner caspases recognize and cleave specific substrates to execute apoptosis (Fan et al., 2005). Calcium homeostasis refers to the balance of Ca^2+^ concentration within and outside cells. Ca^2+^ is one of the most critical intracellular signaling molecules and regulates apoptosis by influencing endoplasmic reticulum and mitochondrial function as well as interactions with Bcl-2 family proteins (Pinton et al., 2008).

miR-873-5p is a microRNA located on human chromosome 9p21.1. Its target genes are closely associated with apoptosis (LIU et al., 2015). For example, miR-873-5p directly targets the 3′-UTR of heme oxygenase-1 (HMOX1) mRNA, thereby regulating HMOX1 expression. HMOX1 is a stress response enzyme closely linked to apoptosis and cognitive impairment (Cressatti et al., 2019). Aqueous extracts of ATR can promote miR-873-5p expression, suppress HMOX1 expression, and reduce apoptosis (Shi et al., 2018). Vascular injuries are major risk factors for dementia. Ischemic damage triggers excessive glutamate release, resulting in Ca^2+^ overload, which consequently leads to abnormal phosphorylation of calcium/calmodulin-dependent protein kinase II (CaMKII) and ultimately neuronal apoptosis and damage (Coultrap et al., 2011). α-Asarone restores the balance of excitatory and inhibitory signals, reduces calcium overload and CaMKII phosphorylation, and inhibits apoptosis, thereby preventing early brain injury following subarachnoid hemorrhage (Gao et al., 2024). CaMKII can phosphorylate critical sites of CREB (Yan et al., 2016), and CREB phosphorylation activates Bcl-2 (Lonze and Ginty, 2002). β-Asarone enhances the expression of CaMKII-α, p-CREB, and Bcl-2, indicating its ability to inhibit neuronal apoptosis via the activation of the CaMKII-α/p-CREB/Bcl-2 pathway (Wei et al., 2013). Apoptosis signal-regulating kinase 1 (ASK1) can be activated by various stress signals and plays a pivotal role in apoptosis. Activated ASK1 subsequently activates mitogen-activated protein kinase kinase 7 (MKK7), which regulates numerous fundamental physiological processes. Under specific stimuli, MKK7 primarily activates JNK (KANG et al., 2024), as previously discussed. Activation of the JNK pathway can promote apoptosis and is closely linked to the onset and progression of dementia. *In vivo* and *in vitro* studies demonstrate that β-Asarone can modulate the ASK1/MKK7/JNK pathway, inhibit the expression of BCL-2-associated X protein (Bax) and Bcl-2 antagonist/killer 1 (Bad), and block the caspase cascade, thereby suppressing apoptosis and protecting neurons (Geng et al., 2010; Zou et al., 2011; Liu et al., 2010; Li et al., 2010).

### 5.5 Modulating neurotransmitters

Neurotransmitters are synthesized and released by presynaptic neurons into the synaptic cleft, where they bind to specific receptors on postsynaptic neurons to modulate their electrical potentials, enabling the transmission of neural signals. Abnormalities in neurotransmission are closely associated with the onset and progression of dementia (Yang et al., 2023). Impairments in the cholinergic system are considered a key factor in learning and memory decline and cognitive dysfunction, forming the basis of the earliest hypothesis regarding the pathogenesis of AD. Severe cholinergic system degeneration in AD patients leads to reduced acetylcholine (ACh) levels, culminating in dementia symptoms (Chen Z. R. et al., 2022). The current first-line drugs for treating dementia, acetylcholinesterase (AChE) inhibitors, are designed based on the cholinergic hypothesis and work by increasing ACh levels in the synaptic cleft to improve cognitive function (Krall et al., 1999). Other neurotransmitters, such as dopamine (DA), glutamic acid (Glu), gamma-aminobutyric acid (GABA), and 5-hydroxytryptamine (5-HT), are also closely associated with learning and memory processes. Their dysfunction contributes to the onset and progression of various dementia subtypes (Duda, 2004; Huey et al., 2006). Research into the role of the volatile oil of ATR in neurotransmitter regulation has primarily focused on these aspects.

ACh is a vital neurotransmitter in the central cholinergic system, and reduced ACh levels are closely linked to the pathogenesis of dementia (Chen Z. R. et al., 2022). AChE is a highly efficient hydrolytic enzyme that catalyzes ACh breakdown into choline and acetate, thereby regulating ACh concentration, whereas choline acetyltransferase (ChAT) is a key enzyme for ACh synthesis. Together, AChE and ChAT maintain the balance of ACh, which is essential for learning and memory (Yang X. X. et al., 2021). α-Asarone reduces AChE activity in the cortex, hippocampus, and striatum of rats, thereby inhibiting ACh hydrolysis, increasing ACh levels, and improving learning and memory function (Venkatesan, 2022). Glu is an excitatory neurotransmitter, while GABA is an inhibitory neurotransmitter. An imbalance between the two may lead to cognitive impairments (Zeydan et al., 2017; Cui et al., 2015). α-Asarone reduces Glu release, improves Glu transport, modulates the overexpression of Glu receptors, and restores the balance between Glu and GABA, thus enhancing cognitive abilities (LI et al., 2019). DA and 5-HT are critical neurotransmitters involved in learning and memory processes, and reductions in DA and 5-HT levels can lead to cognitive impairments (Speranza et al., 2021; Afshar et al., 2019). β-Asarone increases levels of DA metabolites, such as homovanillic acid (HVA) and 3,4-dihydroxyphenylacetic acid (DOPAC), and 5-HT metabolites, such as 5-hydroxyindoleacetic acid (5-HIAA), in the brains of Parkinsonian rats. It also elevates the levels of tyrosine hydroxylase (TH), a key enzyme in the dopamine synthesis pathway (Zhang S. et al., 2016).

### 5.6 Improving synaptic function

Synapses are key hubs for inter-neuronal communication, consisting of the presynaptic membrane, synaptic cleft, and postsynaptic membrane, through which neural information is transmitted sequentially. Synaptic dysfunction is closely associated with numerous neurological disorders. Synaptic impairments, such as synapse loss, dendritic spine damage, and reduced neurotransmission, are often observed in subtypes of dementia, including AD, PD, and VD (Che et al., 2023; Merino-Galán et al., 2022; Piscopo et al., 2022). The number of neurotransmitter receptors and their spatial organization at postsynaptic sites underlie synaptic functions and mediate forms of synaptic plasticity (Groc and Choquet, 2020). Furthermore, neurotrophic factors, especially BDNF, significantly influence synaptic function and structure, playing a crucial role in regulating changes in synaptic structure and functionality (Wang C. S. et al., 2022). Therefore, studies on the improvement of synaptic function by the volatile oil of ATR predominantly focus on these areas.

Ischemic brain injury activates NR2B, leading to intracellular Ca^2+^ influx and subsequent autophosphorylation of CaMKII (Meng et al., 2003). Phosphorylated CaMKII activates cAMP Response Element-Binding Protein (CREB), subsequently promoting BDNF expression. BDNF binds to TrkB receptors, triggering downstream signaling pathways essential for synaptic plasticity regulation (Zhang et al., 2023). α-Asarone enhances the interaction between NR2B and CaMKII, activating the CREB/BDNF/TrkB signaling pathway, thereby increasing hippocampal synapse density and plasticity (Gao et al., 2024). Synaptophysin (SYP), a membrane protein involved in synaptic vesicle fusion and a major component of the presynaptic membrane, correlates closely with cognitive function (Harrill et al., 2015; Xiao et al., 2014). SYP reflects synaptic density and distribution (Sarnat and Born, 1999), and it is thought to enhance neuronal plasticity by influencing synaptic structure and mediating neurotransmitter release through phosphorylation (Chan et al., 2002). Glutamate Receptors (GluRs) mediate excitatory synaptic transmission in the central nervous system, are associated with learning and memory, and play an essential role in synaptic plasticity (Sanderson et al., 2008). SYP primarily facilitates presynaptic neurotransmitter release, while GluR1 predominantly functions at the postsynaptic membrane, mediating signal reception and transmission. Together, they synergistically regulate synaptic strength and structure, impacting learning and memory processes. β-Asarone has been shown to upregulate the expression of SYP and GluR1, thereby modulating synaptic plasticity and mitigating Aβ-mediated neuronal damage (Liu et al., 2016). cAMP (cyclic adenosine monophosphate) is synthesized by Adenylate Cyclase (AC) from ATP. cAMP binds to the regulatory subunits of Protein Kinase A (PKA), releasing the catalytic subunits, which become activated. The activated PKA stimulates the phosphorylation of CREB, a critical transcription factor, which modulates the transcription of downstream proteins such as BDNF to improve synaptic function (Chandía-Cristi et al., 2023). β-Asarone inhibits excessive autophagy, reduces organelle degradation, and enhances mitochondrial energy production, activating the cAMP/PKA/CREB signaling pathway to ameliorate synaptic dysfunction in a mouse model of vascular dementia (Ning et al., 2024). Dendritic spines, primary sites for synaptic contacts, are crucial for receiving convergent synaptic input from other neurons. Abnormalities in dendritic spine morphology and density can contribute to synaptic dysfunction (Rochefort and Konnerth, 2012). Dendritic spine density decreases with aging, and dendritic spine loss is common among dementia patients (Mijalkov et al., 2021). β-Asarone has been observed to restore dendritic spine density in the hippocampal CA1 and DG regions of lead-exposure-induced rats. This is accompanied by increased expression of NR2B, Arc/Arg3.1, and Wnt7a proteins, leading to improved synaptic function (Yang Q. Q. et al., 2016). Among these, NR2B, a subunit of NMDA receptors, is critical for synaptic plasticity and learning and memory processes. Increased NR2B expression enhances synaptic function (Zhuo, 2024). Arc/Arg3.1 is an immediate early gene associated with long-term potentiation (LTP). NMDA receptor activation upregulates Arc/Arg3.1 levels (Messaoudi et al., 2007), while it also triggers Wnt activation within the hippocampus. Wnt promotes presynaptic assembly (Ahmad-Annuar et al., 2006), and Wnt7a, a crucial protein within the Wnt signaling pathway, facilitates synaptic formation and functionality through mechanisms like synapsin1 aggregation and increased miniature excitatory postsynaptic current (mEPSC) frequency (Cerpa et al., 2008).

### 5.7 Regulating autophagy

Autophagy is one of the principal mechanisms maintaining intracellular homeostasis, degrading and recycling damaged or unnecessary components to sustain cellular balance. Dysregulated autophagy has been implicated in the onset and progression of dementia. Impairment of autophagic function reduces the clearance of pathological proteins such as Aβ, p-Tau, and α-synuclein, leading to their aggregation, cytotoxicity, and eventual cell death (Salminen et al., 2013; Winslow and Rubinsztein, 2011). Excessive autophagy, however, is equally detrimental. As disease progresses and abnormal protein aggregates accumulate, sustained activation of autophagy can result in autophagic cell death, exacerbating neuronal loss (Han et al., 2015). The PI3K-Akt-mTOR signaling pathway is central to autophagy regulation. Phosphatidylinositol-3-Hydroxykinase (PI3K) catalyzes phosphatidylinositol phosphorylation to produce Phosphatidylinositol-3,4,5-Triphosphate (PIP3), which accumulates at the cell membrane to recruit and activate Protein Kinase B (Akt). Akt directly phosphorylates and activates mTORC1 (Hou et al., 2018), a key regulator of autophagy-related proteins (Wang and Zhang, 2019). The interaction between Bcl-2 and Beclin-1 is another critical regulatory mechanism. Under physiological conditions, Bcl-2 suppresses autophagy by inhibiting Beclin-1 (Decuypere et al., 2012). Beclin-1 initiates autophagosome formation and recruits other autophagy-related proteins to autophagic vesicles (Wang et al., 2017), making its expression a marker for autophagy activation or inhibition. LC3 and p62 are also considered specific autophagy markers. Microtubule-Associated Protein 1A/1 B-Light Chain 3 (LC3) is intimately involved in autophagosome formation. When catalyzed by Atg4, LC3-I is covalently linked to phosphatidylethanolamine (PE) to form LC3-II, which integrates into autophagosome membranes. Thus, LC3-II levels or the LC3-II/LC3-I ratio reflect autophagy activity and autophagosome quantity (Agrotis et al., 2019). Sequestosome 1(p62), a selective autophagic substrate, bridges ubiquitinated proteins targeted for degradation with LC3 and is similarly indicative of autophagy function (Zhang et al., 2019). Mitophagy—a selective form of autophagy targeting damaged mitochondria—is particularly relevant in AD, where mitochondrial dysfunction contributes to Aβ and Tau pathology, while Aβ and Tau in turn exacerbate mitochondrial dysfunction (Fang et al., 2019). Research on the volatile oil of ATR has predominantly focused on these mechanisms.

PINK1, a serine/threonine kinase localized to depolarized mitochondria, and Parkin, an E3 ubiquitin ligase, are key mediators of the PINK1-Parkin pathway, which facilitates mitophagy (Vincow et al., 2013). β-Asarone significantly increases PINK1 and Parkin expression in the hippocampus of AD rats, reducing Aβ deposition by enhancing mitophagy through the PINK1-Parkin pathway (Han et al., 2020). *In vitro* studies corroborate this, demonstrating that β-Asarone upregulates LC3, Beclin-1, PINK1, and Parkin, while downregulating p62 expression in PC12 cells within AD models, increasing autophagic flux and mitigating Aβ aggregation (Wang et al., 2019). Notably, while autophagosome quantity is traditionally used to assess autophagic activity, it reflects both formation and degradation processes. Impaired autophagosome clearance can lead to increased autophagosome counts, whereas enhanced autophagic flux can reduce their numbers. One *in vivo* study suggests that α-Asarone reduces autophagosome quantities in the hippocampi of APP/PS1 mice, restoring autophagic flux and enhancing autophagy activity, thereby reducing abnormal protein aggregates (Zeng et al., 2021). Another *in vitro* study demonstrated that Aβ inhibits autophagic flux by disrupting autophagosome-lysosome fusion or impairing lysosomal function. β-Asarone restores autophagic flux by reducing autophagosome numbers and increasing autolysosome counts, enhancing autophagosome-lysosome fusion or lysosomal functionality (Li et al., 2021).

Importantly, autophagy does not universally benefit dementia progression. Prolonged autophagy activation may induce neuronal death. α-Asarone lowers LC3-II/LC3-I ratios and upregulates p62 expression, attenuating ischemia-induced excessive autophagy in the rat cortex and protecting neurons (Zhang K. et al., 2021). β-Asarone reduces Beclin-1 and LC3 expression while upregulating p62 in the hippocampi of AD mice (Deng et al., 2020), exhibiting similar effects in vascular dementia models (Ning et al., 2024). Impairments in the PI3K-Akt-mTOR pathway, observed in dementia patients, exacerbate excessive autophagic activation (Rickle et al., 2004). β-Asarone activates the PI3K-Akt-mTOR pathway to inhibit α-synuclein overexpression-induced excessive autophagy in the striatum of Parkinson’s disease model rats (Wang et al., 2024). *In vitro*, β-Asarone similarly reduces Aβ-triggered autophagic overactivation via PI3K-Akt-mTOR pathway activation (Xue et al., 2014). Regulating the interaction between Bcl-2 and Beclin-1 is also pivotal. One *in vitro* study demonstrated that β-Asarone mitigates Aβ_25-35_-induced excessive autophagy via the Bcl-2/Beclin-1 pathway (Chang and Teng, 2015). Additionally, JNK activation downregulates Bcl-2, disrupting its interaction with Beclin-1, thereby promoting autophagy (Trenti et al., 2014). β-Asarone modulates the JNK/Bcl-2/Beclin-1 pathway, reducing JNK and p-JNK expression while increasing Bcl-2 and Beclin-1 levels in the striatum of 6-OHDA-induced Parkinson’s model rats, attenuating excessive autophagy (Zhang S. et al., 2016). Furthermore, misfolded protein accumulation induces endoplasmic reticulum stress (ER stress), activating the Unfolded Protein Response (UPR). While UPR attempts to restore normal ER function, it can also lead to excessive autophagy (Senft and Ronai, 2015), a process common in dementia pathology (Ajoolabady et al., 2022). β-Asarone inhibits the PERK/CHOP/Bcl-2/Beclin-1 pathway to alleviate ER stress and excessive autophagy in Parkinson’s disease rat models, reducing PERK phosphorylation, CHOP expression, and Beclin-1 release, while increasing Bcl-2 levels to inhibit autophagy (Ning et al., 2019).

### 5.8 Mitigating cellular stress

Oxidative stress, characterized by an imbalance between oxidant and antioxidant systems due to excessive accumulation of reactive oxygen species (ROS) and impaired antioxidant defenses, represents a deleterious consequence of free radical production often implicated in aging and disease (Chen and Zhong, 2014). Under physiological conditions, ROS are maintained at low levels and act as signaling molecules to stimulate cell growth. However, under pathological conditions, excessive ROS accumulation can overwhelm antioxidant defenses, resulting in oxidative stress and cellular damage (Halliwell, 2006). The brain, being a high metabolic organ with high oxygen consumption, generates substantial ROS during metabolism. Furthermore, its abundance of unsaturated fatty acids renders it prone to oxidative damage, as they easily undergo lipid peroxidation in the presence of ROS (Radak et al., 2011). Consequently, oxidative stress is closely associated with the onset and progression of dementia. For instance, abnormal proteins like Aβ can induce oxidative stress by affecting mitochondrial function and metal ion homeostasis (Pagani and Eckert, 2011; Sohrabi et al., 2023; Huang et al., 1999). Both acute and chronic hypoxia have been found to mediate oxidative stress (Snyder et al., 2017). In conclusion, oxidative stress represents an early pathological event in the brains of dementia patients, where increased levels of lipid peroxidation products and decreased antioxidant enzyme activities are often observed (Chen J. et al., 2022; Atiq et al., 2023). Endoplasmic reticulum (ER) stress is another pivotal factor in cellular stress that is closely associated with the pathogenesis of dementia. ER stress arises when protein folding or modification in the ER becomes dysregulated, triggering an unfolded protein response (UPR). The UPR, a primary adaptive mechanism against ER stress, is mediated by three major signaling pathways involving the ER stress sensors IRE1α, PERK, and ATF6. Under normal conditions, these sensors are found bound to the ER chaperone GRP78, maintaining them in an inactive state (Todd et al., 2008). Upon accumulation of misfolded or unfolded proteins, GRP78 preferentially binds to the abnormal proteins and dissociates from PERK, ATF6, and IRE1α, leading to the activation of these ER stress sensors (Hotamisligil, 2010). Mirroring oxidative stress, ER stress is a critical factor in the development of dementia (Jiang et al., 2010).

The primary defense mechanism against ROS involves the elimination of ROS by antioxidant enzymes such as superoxide dismutase (SOD), glutathione peroxidase (GPx), and catalase (CAT) (Jomova et al., 2024). A secondary line of defense involves uncoupling proteins (UCPs), which restrict excessive mitochondrial ROS production (Hass and Barnstable, 2021). The volatile oil of ATR has been shown to enhance CREB phosphorylation at Ser-133, thereby activating peroxisome proliferator-activated receptor gamma coactivator 1-alpha (PGC-1α) and upregulating SOD, GPx1, and UCP to counteract H_2_O_2_-induced oxidative stress in PC12 cells (Yan et al., 2020b). α-Asarone reduces ROS levels (Mikami et al., 2021), while β-Asarone elevates hippocampal SOD and GPx levels in AD rat models, reducing oxidative stress and neuronal injury (Saki et al., 2020). Further studies have demonstrated that the volatile oil of ATR, α-Asarone, and β-Asarone activate the Akt signaling pathway, inducing Antioxidant Response Element (ARE) transcriptional activation and upregulating antioxidant enzymes such as glutathione S-transferase (GST) and NAD(P)H quinone oxidoreductase 1 (NQO1), which facilitate ROS elimination (Lam et al., 2017b). β-Asarone promotes the phosphorylation of PI3K/Akt, enhancing the expression of NF-E2-related factor 2 (Nrf2) and heme oxygenase-1 (HO-1) (Meng et al., 2021). Activated Nrf2 dissociates from kelch-like ECH-associated protein 1 (Keap1), translocates to the nucleus, and binds to ARE, activating downstream genes coding for detoxifying enzymes and antioxidants like HO-1 (Raghunath et al., 2018). HO-1, which is positively regulated by Nrf2, increases GSH-Px, SOD, and CAT expression to improve antioxidant capacity (Ryter, 2021). Additionally, Aβ induces excessive nitric oxide (NO) production via nitric oxide synthase (NOS), and NO, as a free radical, participates in lipid peroxidation by forming peroxynitrite anions (Limón et al., 2009b). α-Asarone has been shown to reduce excessive NO levels in Aβ-induced hippocampal and temporal cortex injury in rats (Limón et al., 2009a). *In vitro* studies corroborate this, as α-Asarone and β-Asarone lower Aβ25–35-induced NO and inducible NOS (iNOS) levels in PC12 cells (Shi et al., 2021).

Regarding ER stress, PERK phosphorylates eukaryotic initiation factor 2 alpha (eIF2α) (Shi et al., 1998), with eIF2α enhancing Activating Transcription Factor 4 (ATF4) translation, subsequently promoting CHOP transcription (Zinszner et al., 1998). α-Asarone inhibits PERK phosphorylation and tunicamycin-induced ER stress (Mikami et al., 2021). β-Asarone exhibits similar effects by reducing GRP78, p-PERK, and CHOP expression at both mRNA and protein levels in the striatum of Parkinson’s disease rats, thereby mitigating ER stress and autophagy via the PERK/eIF2α pathway (Ning et al., 2019). Likewise, IRE1α, another key mediator of ER stress, undergoes autophosphorylation and oligomerization after dissociation from GRP78, resulting in the splicing of XBP1 mRNA to generate XBP1s, which degrades unfolded proteins (Li Y. et al., 2024). In Parkinson’s disease animal models, β-Asarone acts as an IRE1 inhibitor, modulating ER stress through the IRE1/XBP1 pathway (Ning et al., 2016).

### 5.9 Inhibiting neuroinflammation

Neuroinflammation, a defensive response occurring within neural tissue, is closely linked to the onset and progression of dementia. Even at early stages of dementia, neuroinflammation—primarily mediated by microglial and astroglial cells—is detectable within the brain (Ahmad et al., 2022). Under normal conditions, these cells aid in clearing abnormal proteins and repairing neurons (Mcalpine et al., 2021). However, during advanced dementia, persistent glial cell activation results in the release of significant levels of inflammatory cytokines, exacerbating protein deposition and leading to neurotoxicity (Hoozemans et al., 2011). Pathogen-associated molecular patterns (PAMPs) or danger-associated molecular patterns (DAMPs) are recognized by pattern recognition receptors (PRRs), activating the IKK complex. This complex phosphorylates and degrades IκB, releasing nuclear factor kappa-B (NF-κB), which activates downstream production of inflammatory cytokines like TNF-α and IL-6 and upregulates NOD-like receptor thermal protein domain-associated protein 3 (NLRP3) expression (Kawasaki and Kawai, 2014). Activation of NLRP3 recruits ASC and pro-caspase-1, forming an inflammasome complex that activates caspase-1 (Zhan et al., 2022). Caspase-1 cleaves gasdermin-D (GSDMD) into GSDMD-N and GSDMD-C fragments, with GSDMD-N forming membrane pores (Meng et al., 2023), while simultaneously processing pro-IL-1β and pro-IL-18 into active IL-1β and IL-18, which are released to promote inflammation (Qin and Zhao, 2023). Pro-inflammatory cytokines like TNF-α and IL-1β further sustain NF-κB activation, creating a vicious cycle (Yu et al., 2020).

The volatile oil of ATR suppresses hippocampal NF-κB and IKKβ phosphorylation in 3×Tg-AD mice, reducing NLRP3 inflammasome activation and downregulating ASC, caspase-1, and the level of GSDMD-N, thereby exerting neuroprotective effects (Xu et al., 2023). Ionized calcium-binding adapter molecule 1 (Iba-1) and glial fibrillary acidic protein (GFAP) are markers of microglial and astroglial activation. α-Asarone decreases GFAP levels and suppresses astroglial activation in APP/PS1 mouse hippocampi, alongside reducing pro-inflammatory cytokines TNF-α, IL-1β, and IL-6 (Zeng et al., 2021). Similarly, β-Asarone decreases GFAP, IL-1β, and TNF-α levels in the brains of AD rats (Yang et al., 2017) and reduces Aβ_25–35_-induced mRNA levels of IL-6, IL-1β, and TNF-α in SH-SY5Y cells, mitigating both neuroinflammation and inflammation-induced autophagy (Chang and Teng, 2015). Both α-Asarone and β-Asarone inhibit microglial activation by reducing IκB-α phosphorylation and degradation, blocking NF-κB activation, and downregulating pro-inflammatory cytokines TNF-α, IL-1β, and IL-6. *In vitro*, α-Asarone decreases markers of microglial-mediated inflammation, including Mac-1, CD-68, Iba-1, iNOS, and COX-2 (Kim et al., 2015). The MAPK signaling pathway also contributes to microglial activation. α-Asarone and β-Asarone downregulate JNK and p38MAPK, reducing pro-inflammatory cytokine levels while increasing anti-inflammatory cytokine IL-10 expression via inhibition of the JNK/MAPK pathway (Shi et al., 2021).

### 5.10 Mitigating vascular risk factors

Cerebrovascular damage, characterized by anatomical disruptions via vascular pathology, is a major cause of vascular cognitive impairment and dementia (VCID). Increasing evidence also implicates vascular risk factors in the progression of various dementia subtypes. Pathological changes, including atherosclerosis, cerebral amyloid angiopathy, and venous collagenopathy, not only contribute to vascular lumen narrowing but also disrupt cerebral autoregulation, reducing cerebral blood flow (Yu et al., 2022). Ischemia and resulting hypoxia induce oxidative stress, endothelial dysfunction, neuroinflammation, neuronal apoptosis, autophagy, and BBB impairment, promoting dementia progression (Burtscher et al., 2021). For example, hypoxia disrupts mitochondrial mechanisms, reduces astrocytic glutamate reuptake, induces glutamate-mediated toxicity, and alters vascular-specific gene expression, resulting in endothelial dysfunction. Vascular endothelial damage in the substantia nigra, brain stem and cerebral cortex was detected in both MPTP-induced animal models and brain samples from PD patients (Sarkar et al., 2014). Furthermore, ischemia and hypoxia often result in impaired cerebrovascular reactivity (CVR), which is linked to dementia, including AD and PD, as well as MCI-to-dementia progression (Camargo et al., 2015; Buratti et al., 2015). In addition, damage to the BBB is one of the core mechanisms and pathological features of cerebrovascular disease (Gao Y. et al., 2022). When BBB integrity is damaged, harmful exudates and inflammatory factors enter the brain tissue, accelerating neuronal damage (Knox et al., 2022), and also leading to reduced Aβ drainage in the perivascular space, resulting in abnormal accumulation of Aβ in the brain (Thal, 2009). In summary, vascular damage and aging are vital contributors to dementia pathogenesis (Andersson and Stone, 2023), as evidenced across diverse dementia subtypes (Raz et al., 2016). Thus, reducing vascular risk factors is a promising strategy for dementia prevention and treatment.

Atherosclerosis is a major cause of reduced cerebral perfusion and hypoxia. α-Asarone has been shown to block endoplasmic reticulum (ER) stress-mediated macrophage apoptosis via interference with caspase-12, IRE1α, and PERK (Park et al., 2016), indicating its potential to antagonize arteriosclerosis and ameliorate the vascular risk factors associated with dementia. Ischemic injury triggers excessive glutamate release, leading to Ca^2+^ overload, which in turn induces abnormal phosphorylation of calcium/calmodulin-dependent protein kinase II (CaMKII), ultimately causing neuronal apoptosis and damage (Coultrap et al., 2011). α-Asarone has been found to restore excitatory-inhibitory balance, reduce calcium overload and CaMKII phosphorylation, and inhibit apoptosis, thereby preventing early neuronal injury following subarachnoid hemorrhage (Gao et al., 2024). Furthermore, α-Asarone mitigates cerebral infarct volume, improves neurological function, reduces GFAP and Iba-1 expression, inhibits glial activation, suppresses autophagy as evidenced by the reduced LC3II/LC3I ratio and increased p62 expression, and lowers the risk of ischemia-reperfusion-induced neuronal injury (Zhang K. et al., 2021). β-Asarone enhances the learning and memory abilities of AD model rats, improves relative cerebral blood flow (rCBF), reduces lactate and pyruvate levels in brain tissue, increases Na^+^-K^+^-ATPase activity, and downregulates hippocampal ET-1 mRNA expression, exhibiting vascular protective effects in AD rats (Li et al., 2012). An increasing body of evidence indicates that oxidative stress plays a central role in ischemia-reperfusion injury (Jakesevic et al., 2011). β-Asarone reduces the risk of localized cerebral ischemia-reperfusion injury and improves neurological function by enhancing the activity of enzymes involved in oxidative stress regulation, including lactate dehydrogenase (LDH), glutathione reductase (GR), catalase (CAT), glutathione S-transferase (GST), glutathione (GSH), glutathione peroxidase (GPx), and Na^+^-K^+^-ATPase (Yang et al., 2013). The Nrf2-ARE pathway not only regulates the transcription of numerous antioxidant genes but also modulates apoptosis, making the activation of Nrf2 and its downstream genes a promising strategy for preventing ischemia-reperfusion-induced neural injury (Shi et al., 2019). β-Asarone activates the Nrf2-ARE pathway, enhancing the expression of Nrf2, NQO1, GCLM, HO-1, and GCLC proteins, inhibiting cortical neuronal apoptosis, reducing cerebral infarct volume in MCAO rats, and improving neurological outcomes (Pan et al., 2021). Oxidative stress can also act as a signal for inducing autophagy (Redza-Dutordoir and Averill-Bates, 2021), forming a vicious cycle. Through modulation of the cAMP/PKA/CREB signaling pathway, β-Asarone reduces oxidative stress levels, suppresses excessive autophagy, restores mitochondrial function, and alleviates synaptic dysfunction and brain tissue damage in vascular dementia mice (Ning et al., 2024). Additionally, β-Asarone regulates the JNK/Bcl-2/Beclin-1 pathway, decreasing JNK and p-JNK expression while increasing Bcl-2 levels to modulate Beclin-1, thereby suppressing ischemia-reperfusion-induced excessive autophagy in brain tissue (Liu et al., 2012). As previously discussed, endothelial dysfunction plays a pivotal role in linking cerebrovascular disease and dementia. Endothelial dysfunction reduces NO bioavailability and inactivates endothelial nitric oxide synthase (eNOS) in cerebral microvascular endothelial cells, consequently upregulating amyloid precursor protein and its cleavage enzymes, ultimately leading to Aβ deposition (Koizumi et al., 2016). β-Asarone increases the number of CD31 and Ki-67-positive cells, promotes VEGFA expression, and upregulates eNOS levels in MCAO model rats (Sun et al., 2024), suggesting its potential to alleviate endothelial dysfunction and promote angiogenesis.

## 6 Discussion

Advancements in dementia research increasingly reveal that the underlying mechanisms of the disease’s pathogenesis are complex, involving the interplay of multiple factors rather than a singular cause. In various subtypes of dementia, interactions between processes such as abnormal protein aggregation, disrupted neurogenesis, autophagy dysregulation, neuronal apoptosis, cellular stress, synaptic dysfunction, neurotransmitter imbalances, neuroinflammation, and vascular damage create a vicious cycle that collectively drives disease progression. Furthermore, a specific pathological mechanism may contribute to multiple dementia subtypes, and the boundaries between these subtypes are not always distinct. For instance, while Aβ has long been associated with AD, it is also observed in PD and DLB (Sengupta and Kayed, 2022), as well as in the brains of VD patients, particularly in cerebral vessel walls, a condition termed cerebral amyloid angiopathy (CAA) (Kalaria, 2003). Numerous similar examples exist, and given the complexity of the disease’s mechanisms, single-mechanism therapies may be difficult to achieve substantial outcomes. Currently, the mitigation of dementia primarily depend on synthetic chemical drugs. However, the narrow therapeutic spectrum and singular mechanism of action are common limitations of these treatments. Conversely, the plant metabolites from medicinal plants are easily accessible, cost-effective, and have been employed for disease management throughout history. In recent years, a growing number of the plant metabolites have been found to exhibit analgesic, sedative, antidepressant, anticonvulsant, antiepileptic, and neuroprotective effects, demonstrating a distinct advantage (Zhang Y. et al., 2021).

Compared to conventional drugs, the volatile oil of ATR contains multiple metabolites targeting various pathways and mechanisms, which may potentially produce synergistic or additive effects. Currently, α-Asarone and β-Asarone are widely recognized as the main active ingredients of the volatile oil of ATR in mitigating dementia. Consequently, much of the current research focuses on testing drugs containing α-Asarone and β-Asarone, while studies exploring the combined effects of the volatile oil of ATR or other metabolites within it in mitigating dementia remain rare. This scarcity may be attributed to the challenges posed by studying medicinal plant extracts in pharmacological research. However, this also raises the question of whether there are synergistic responses among different metabolites in the process of mitigating dementia using the volatile oil of ATR. For instance, some researchers have proposed that α-Asarone and β-Asarone, in their natural ratio (1:4) as present in the volatile oil of ATR, exhibit synergistic effects in enhancing neurofilament promoter activity while reducing side effects (Lam et al., 2016b). Does a similar synergistic effect also exist among other metabolites in the volatile oil of ATR, even those present in relatively low abundance? This hypothesis may serve as one of the potential directions for further investigation in the future. Additionally, the high lipophilicity of the volatile oil of ATR facilitates its ability to cross the BBB, overcoming the challenges faced by many drugs in infiltrating brain tissue. However, as previously mentioned, while current studies generally agree that the volatile oil of ATR can traverse the BBB, there is ongoing debate regarding whether it repairs BBB damage or simply increases BBB permeability. Such discrepancies highlight the need for further in-depth research into the specific mechanisms by which the volatile oil of ATR or its main ingredients interacts with the BBB, in order to determine whether they have potential side effects. At the same time, it must be noted that while the volatile oil of ATR have demonstrated promising anti-dementia effects and are generally considered a safe traditional medicine, as previously mentioned, there may still be potential toxic side effects. To mitigate toxicity, appropriate dosage selection and treatment duration should be carefully considered. However, the volatile oil of ATR faces the challenge of low oral bioavailability, which presents a disadvantage for treatment regimens that require high doses or long-term administration. As noted earlier, advancements in delivery systems (Inamdar et al., 2024), such as nanoparticle delivery and intranasal delivery, can significantly enhance the bioavailability of the drug (Ahmad et al., 2018). Therefore, the issues of low bioavailability and potential toxic side effects associated with the volatile oil of ATR may be addressed or avoided through these improvements. Nonetheless, research on the administration routes of the volatile oil of ATR is currently very limited, and the extent to which different administration methods affect its safety requires further evaluation. Moreover, numerous recent studies on the volatile oil of ATR lack standardized experimental methodologies. For instance, significant variations exist in the extraction methods of the volatile oil of ATR, dosage selection, routes of administration (such as oral, inhalation, or injection), and experimental durations, making it challenging to compare or integrate the findings. While such inconsistencies may stem from the progressive nature of dementia as well as the inherent challenges of studying medicinal plant extracts in pharmacological research, the need for standardized and systematic studies remains paramount. Furthermore, although the volatile oil of ATR has a long history of medicinal use, the clinical efficacy and safety of botanical drugs and plant-derived metabolites should not be generalized. Current research primarily focuses on pharmacological studies, with evidence mostly derived from molecular mechanism studies in cell and animal models. Systematic preclinical toxicity studies remain lacking, and large-scale, long-term clinical trials using the volatile oil of ATR as a standalone therapy are scarce. Thus, systematic investigations into its efficacy and safety are urgently needed.

For future research, the following questions can be explored, but are not limited to these aspects: (1) Do other less abundant metabolites in the volatile oil of ATR play a role in dementia intervention? Are there synergistic or additive effects among these metabolites? (2) What is the mechanism by which the volatile oil of ATR penetrates the BBB? Does it increase BBB permeability and, if so, could this result in unintended side effects? (3) Does the efficacy of the volatile oil of ATR in dementia mitigation vary significantly depending on extraction methods, dosage, administration routes, and experiment duration? Is it possible to establish an optimized and standardized protocol for its extraction and application? (4) How can the safety of the volatile oil of ATR under long-term use or high dosages be determined? What internationally accepted standardized toxicological studies can be employed to comprehensively evaluate its safety? (5) Can optimizing drug delivery systems—such as nanoparticles or intranasal administration—significantly enhance the bioavailability of the volatile oil of ATR? Could these innovative delivery systems alter its efficacy, safety, and pharmacokinetic properties? (6) Are there significant differences in the mechanisms of action and efficacy of the volatile oil of ATR in preventing dementia during the cognitive impairment stage versus treating it during the dementia stage? (7) Can the extensive mechanistic studies conducted on cellular and animal models be effectively translated into systematic clinical research to verify its efficacy and safety?

Overall, the volatile oil of ATR demonstrates significant potential in alleviating dementia. While current research provides a relatively comprehensive understanding of its mechanisms of action in dementia intervention, further foundational studies and clinical research are required to work in tandem. Employing standardized and systematic approaches is essential to overcome critical challenges and ultimately achieve robust validation of its efficacy and safe application.

## 7 Conclusion

In conclusion, the multi-pathway, coordinated pharmacological effects of the volatile oil of ATR align well with the complex mechanisms underlying the development of dementia. This highlights its significant potential in mitigating dementia and presents a promising opportunity for the treatment of not only dementia but also other neurodegenerative diseases. This potential could pave new avenues for future research. Nonetheless, further rigorous and high-quality studies are required to overcome the associated challenges, including ensuring the accuracy and stability of the chemical characterization of the volatile oil of ATR, clarifying the synergistic interactions between its metabolites, identifying potential toxicities, and validating its exact clinical efficacy.
